# Pediatric tele-coaching fidelity evaluation: Feasibility, perceived satisfaction and usefulness of a new measure

**DOI:** 10.3389/fresc.2023.1057641

**Published:** 2023-02-21

**Authors:** Tatiana Ogourtsova, Annette Majnemer, Amelie Brown, Helen Jillian Filliter, Kristy Wittmeier, Jessica Hanson, Maureen O’Donnell

**Affiliations:** ^1^ Jewish Rehabilitation Hospital, Laval, QC, Canada; ^2^ Faculty of Medicine, School of Physical and Occupational Therapy, McGill University, Montreal, QC, Canada; ^3^ Centre for Interdisciplinary Research in Rehabilitation of Greater Montreal (CRIR), Montreal, QC, Canada; ^4^ Montreal Children’s Hospital, Research Institute of the McGill University Health Centre, Montreal, QC, Canada; ^5^ Department of Pediatrics, Dalhousie University, Halifax, NS, Canada; ^6^ Autism Team, IWK Health Centre, Halifax, NS, Canada; ^7^ Rehabilitation Centre for Children, Winnipeg, MB, Canada; ^8^ Department of Pediatrics and Child Health Rady Faculty of Health Sciences, University of Manitoba, Winnipeg, MB, Canada; ^9^ Children’s Hospital Research Institute of Manitoba, Winnipeg, MB, Canada; ^10^ Provincial Health Services Authority BC, Vancouver, BC, Canada; ^11^ Faculty of Medicine, Department of Pediatrics, University of British Columbia, Vancouver, BC, Canada

**Keywords:** coaching, childhood disability, coaching fidelity, developmental disability, health coaching

## Abstract

**Background:**

To promote and ensure coaches' fidelity in delivering an online health coaching program to parents of children with suspected developmental delay, we developed and implemented a novel coaching fidelity rating tool, CO-FIDEL (COaches Fidelity in Intervention DELivery). We aimed to (1) Demonstrate CO-FIDEL's feasibility in evaluating coaches' fidelity and its change over time; and (2) Explore coaches' satisfaction with and usefulness of the tool.

**Methods:**

In an observational study design, coaches (*n* = 4) were assessed using the CO-FIDEL following each coaching session (*n* = 13–14 sessions/parent-participant) during the pilot phase of a large randomized clinical trial involving eleven (*n* = 11) parent-participants. Outcome measures included subsections' fidelity measures, overall coaching fidelity, and coaching fidelity changes over time analyzed using descriptive and non-parametric statistics. In addition, using a four-point Likert Scale and open-ended questions, coaches were surveyed on their satisfaction and preference levels, as well as facilitators, barriers, and impacts related to the use of CO-FIDEL. These were analyzed using descriptive statistics and content analysis.

**Results:**

One hundred and thirty-nine (*n* = 139) coaching sessions were evaluated with the CO-FIDEL. On average, overall fidelity was high (88.0 ± 6.3 to 99.5 ± 0.8%). Four coaching sessions were needed to achieve and maintain a ≥ 85.0% fidelity in all four sections of the tool. Two coaches showed significant improvements in their coaching skills over time in some of the CO-FIDEL sections (Coach B/Section 1/between parent-participant B1 and B3: 89.9 ± 4.6 vs. 98.5 ± 2.6, *Z* = −2.74, *p* = 0.00596; Coach C/Section 4/between parent-participant C1 and C2: 82.4 ± 7.5 vs. 89.1 ± 4.1, *Z* = −2.66; *p* = 0.00758), and in overall fidelity (Coach C, between parent-participant C1 and C2: 88.67 ± 6.32 vs. 94.53 ± 1.23, Z = −2.66; *p* = 0. 00758). Coaches mainly reported moderate-high satisfaction with and usefulness of the tool, and pointed out areas of improvement (e.g., ceiling effect, missing elements).

**Conclusions:**

A new tool ascertaining coaches' fidelity was developed, applied, and shown to be feasible. Future research should address the identified challenges and examine the psychometric properties of the CO-FIDEL.

## 1. Introduction

Caregivers of children with or with suspected developmental disabilities experience heightened levels of stress, other psychosocial and physical health issues, and changes to family dynamics (1). Furthermore, families may face different challenges while navigating the healthcare system, including long waiting periods, service gaps, and duplication in services (2). Health coaching is a method that recently emerged to address issues in a rising number of children and families in need, designed to promote caregiver self-management and their sense of empowerment in such commonly demanding yet potentially modifiable situations (3). Health coaching is an educational, structured program that can be delivered by more accessible and potentially cost-effective means (e.g., telephone, online). It is a “goal-oriented, client-centered partnership that is health-focused and occurs through a process of client-enlightenment and empowerment” (4) (p. 24). In the context of childhood disability, a coach delivering the program encourages parental learning through the development of collaborative partnership, guiding the parent to attain self-determined objectives using various learning techniques, and elaborating on the parent's existing competencies (5, 6).

*BRIGHT Coaching* is a tele-coaching program designed to empower and support caregivers of children with emerging developmental delays who are waiting for services in relation to their child's needs. It is currently being trialed in a nationwide randomized clinical study, across four Canadian provinces, including British Columbia, Manitoba, Quebec, and Nova Scotia (3). *BRIGHT Coaching* is delivered by four coaches, one in each participating province. The program is composed of twelve coaching topics (Supplementary Material S1) and incorporates key elements of coaching in early childhood (e.g., non-directive, goal-oriented, solution-focused, reflective, collaborative approach) (5). As *BRIGHT Coaching* is offered across four Canadian provinces by different coaches, it was essential to ensure that coaches' skills align with the intervention goals (i.e., extent to which core components of the intervention are delivered as intended by protocol) and are comparable among coaches. A fidelity assessment procedure to ensure faithfulness of the coach approach to the program requirements is suggested to be implemented for accurate interpretation of treatment effects in intervention-based research (7, 8), justifying and increasing the validity of findings. Gearing et al. (2011) identified four core components of a treatment program fidelity: (1) Intervention Design and Protocol; (2) Intervention Training; (3) Monitoring of Intervention Delivery; and (4) Monitoring of Intervention Receipt. The third component—Monitoring of Intervention Delivery—is viewed as the essence of treatment fidelity and encompasses fidelity assessment during the treatment program (8).

Provided that a pediatric health coaching approach delivered online is a novel approach, assessing coaching fidelity for these kinds of interventions remains in its infancy. Moreover, existing evaluation methods are not optimal. For instance, the *Coaching-in-Action Checklist for Fidelity Assessment* (9), which is used in early intervention coaching programs, is based on a dichotomous yes/no rating format and does not offer an overall score or individual sections' scores. Hence, it limits response variability for the rater, the ensuing interpretation of findings, and its usefulness in determining baseline levels and changes over time (e.g., maintenance, improvement, or deterioration of coaching skills). Similarly, although the *Coaching Practices Rating Scale* (10) is based on a five-point Likert scale, it does not offer an overall score or individual sections' scores for different elements of coaching. Moreover, this tool is self-administered, introducing subjectivity and possibly resulting in response bias. Other existing coaching interventions in childhood disability (e.g., *Coaching in Context* (11)) do not offer standardized fidelity assessment methods.

In view of these existing limitations and practice gaps, our team sought to develop and implement a new intervention-specific coaching fidelity rating tool for *BRIGHT Coaching*: CO-FIDEL (**CO**aches **F**idelity in **I**ntervention **DEL**ivery). In line with the Comprehensive Intervention Fidelity Guide Component 3 – Monitoring Intervention Delivery (12), our objectives were to demonstrate CO-FIDEL's feasibility in evaluating coaches' fidelity and its change over time during *the BRIGHT Coaching* randomized clinical trial pilot phase (Objective 1). Moreover, we sough to explore coaches' satisfaction with and usefulness of the tool (Objective 2).

## 2. Methods

### 2.1. CO-FIDEL development process

CO-FIDEL's development process (Supplementary Material S2) included two approaches: (1) An opinion-seeking technique from experts in the field of coaching and childhood disability; and (2) A rapid literature review of the intervention studies related to coaching for their coaches' fidelity ascertainment procedures. Four (*n* = 4) experts in the field of family coaching and/or childhood disability research and clinical practice participated to CO-FIDEL development: a Social Worker & Psychotherapist (AB); Researchers (Occupational Therapist (AM); Developmental Pediatrician (MO)); and a Postdoctoral fellow—Occupational Therapist (TO).

In the scope of *BRIGHT Coaching*, our team conducted and published a systematic review and analysis on existing coaching interventions that are provided to parents of children with or with suspected developmental disabilities (3). This systematic review included 28 intervention studies. All of them were part of the rapid review process, extracting information on coaches training and fidelity ascertainment, where present. Findings were then presented to the team, generating discussion on the features of an optimal coaching fidelity evaluation tool: content, nature, administration method, frequency of administration, and scoring. In relation to *BRIGHT Coaching* and the childhood disability coaching principles, the following elements, reflecting intervention fidelity, were considered in the CO-FIDEL's design:

• *Types of behaviors to be measured*: behaviors that are program-specific, essential, and also behaviors that need to be avoided (12).
• *Coaching competence*: the level of engagement with participant (13), and the sensitivity with which the treatment protocol was applied (14).
• *Measures*: Frequency counts of particular behaviors (12) and use of a rating scale to better reflect rater's true evaluation (15).

Only six out of the 28 included studies in our systematic review (3) referred to the ascertainment of coaches' fidelity in delivering their health coaching program to parents of children with developmental disabilities (16–21). Table S1 (Supplementary Material S3) outlines the extracted data. Overall, the fidelity assessment procedures in terms of content and methods were not described in enough detail. The method of assessment was either a review of an audiotaped or a videotaped session or supervision of active cases by the principal investigator or an accredited practitioner. Evaluation frequency ranged from weekly to bi-monthly. Only one study specified that coaches needed to attain a fidelity score of >90% to begin the provision of the intervention to study participants (17).

Following the review of these findings and a team discussion among experts in the fields of coaching and childhood disability, we decided to incorporate principles of the Motivational Interview Skills Code (MISC) (22, 23) and the Solution-Focused Interview Skills (SFIS) (24, 25) to the rating tool. MISC refers to demonstrating skills related to acceptance, empathy, and spirt, including collaboration, evocation, and autonomy support. Consistent MISC responses included the following: advise with permission, affirm, emphasize control, question openly, reflect, reframe, and support. Inconsistent responses included: advise without permission, confront, direct, raise concern without permission and warn (22, 23). SFIS refers to using open-ended questions, summaries, tolerating and using silences, complimenting participants' strengths and past/current successes, and affirming client's perceptions (24, 25). Given that our coaches were trained on employing those two techniques within their *BRIGHT Coaching* sessions, it was deemed appropriate to evaluate their performance based on these concepts. In addition, we incorporated a section on the overall ability of the coach to deliver the content and their attitude during the session. Following the initial development of CO-FIDEL in close collaboration between the four experts, a draft version of the tool was presented to the participating coaches for their feedback and adjusted accordingly prior to implementation.

As a result, Version 1.0 of the CO-FIDEL contains 4 ratable sections*.* The cover page includes descriptors of the evaluated session and dedicated space for general comments (Supplementary Materials S4 and S5). Sections 2, 3, and 4 of the tool were designed to match the types of behaviors to be measured that are program-specific, essential, and behaviors that need to be avoided (12). In addition, as recommended by previous research, we considered including frequency counts of particular behaviors (12) (i.e., Section 3) and use of a rating scale to better reflect rater's true evaluation (15) (i.e., Sections 1, 2 and 4).

### 2.2. Coaches training in providing the BRIGHT coaching program

A registered health professional, who is an experienced social worker and family counselor with expertise in family/child counseling, held the role of the Lead Coach. Prior to launching the trial's pilot phase, the Lead Coach provided training to the four coaches. The hybrid training was delivered face-to-face and online in different formats, including individual, group activities, and learning workshops using a case-study approach. The main objectives of the training were to fortify coaches' skills in motivational (22, 23) and solution-focused (24, 25) interviewing techniques, individual and collaborative goal-setting, and shared decision-making in relation to the *BRIGHT Coaching* topics. Motivational and solution focused interviewing skills are applied by the coaches throughout the *BRIGHT Coaching* sessions to encourage parents' autonomy in decision-making and finding lasting solutions to issues as they witness their child's developmental challenges emerge. Using these two approaches, coaches act as guides, and actively engage parents. Coaches evoke and elicit parents' strengths and aspirations, listen to and work through their concerns, boosting their confidence in their ability for positive change.

Coaches received supporting training materials to deliver the intervention, including the Coach Manual. This manual outlines the coaching topics and contains cues and prompts to promote active engagement and participant's reflection. Moreover, to stimulate iterative training and support coaches' skills throughout the trial, ongoing training activities were organized and delivered by the Lead Coach in the form of individual/group discussions, experiential learning and sharing of best practices. Regular activities included bi-weekly individual meetings between the Lead Coach and each coach and weekly group meetings between the Lead Coach and all coaches. Continuing collaboration check-ins between the Lead Coach and participating coaches supported *BRIGHT Coaching* delivery.

### 2.3. Study design

To address the study objectives, an observational study design was used.

### 2.4. Participants

#### 2.4.1. Coaches

Four (*n* = 4) coaches were involved. All were new to the *BRIGHT Coaching* program. Coaches had on average 12.8 ± 6.5 years of experience in the field of social work, family functioning, family support, and/or child development. At the time of the trial, for forthcoming real-life generalizability purposes, three of the four coaches were not registered health professionals.

#### 2.4.2. Parent-participants

Parent-participants were included in the pilot phase of the randomized clinical trial based on the following eligibility criteria: being a caregiver to a child (minimum age 1.5 years old) who is referred for diagnosis and/or therapeutic interventions due to emerging delay(s) in one or more domains (e.g., motor, cognitive, speech, social and/or behavioral); having regular access to the Internet using a desktop, laptop, or a mobile device; being comfortable talking and reading in English or in French.

All coaches and parent-participants have provided free and informed consent to participate in the study.

### 2.5. Study procedures

#### 2.5.1. Objective 1

As part of the *BRIGHT Coaching* randomized clinical trial's pilot phase, coaches were working with a minimum of one parent-participant, while some coaches had begun to work with several participants. The Lead Coach measured coaches’ fidelity following each coaching session using the CO-FIDEL by listening to the recording of the coaching session. The results were then shared with each coach and reviewed by the Lead Coach before their next scheduled coaching session. Outcomes included the fidelity rating in each of the four sections of CO-FIDEL; the overall fidelity rating; the number of sessions needed to achieve and maintain ≥85% fidelity rating (as per previously established acceptable level (7)) in each section of the CO-FIDEL; the individual scores change over time; and the scores differences between coaches.

#### 2.5.2. Objective 2

A self-administered questionnaire consisting of a series of statements to be rated using a four-point Likert-Scale (ranging from “Not at all” – “Very much”) and open-ended questions was used with the coaches (Supplementary Material S6). This questionnaire was designed in-house and administered to the participating coaches at the end of the pilot phase. Outcomes included satisfaction with CO-FIDEL's content, format, administration frequency, understandability, comprehensiveness, relevance, usefulness, and coaches’ perspectives of the benefits and challenges of using this tool.

### 2.6. Measurement and data analysis

#### 2.6.1. Objective 1

Version 1.0 of the CO-FIDEL contains four ratable sections (detailed description and rating procedures found in Supplementary materials S4 and S5):

(1) ***Overview: Coach Delivering Content & Attitude***: Refers to the coach's ability to guide the participants through the content of each session, respecting the timeline and the related activities of each session, and the overall attitude of the coach during the session (e.g., ability to balance between spontaneous counseling and *BRIGHT Coaching* manual content). This section was designed to parallel with the basic coaching competencies needed to be measured (13, 14). It has ten performance factors, where each is scored on a 7-point scale ranging from 1 - “Low ability” to 7- “High ability”. An overall percentage score (ranging from 14.3–100) is obtained for this section as follows:

$$\frac{\sum \mathrm{scores}\;\mathrm{in}\;\mathrm{each}\;\;\mathrm{of}\;\mathrm{the}\;10\;\mathrm{subsections}}{70}\times \;100$$

(2) ***Global Coach Ratings/Motivational Interviewing Skills Code (MISC)****:* Refers to the coach's motivational interviewing skills (e.g., acceptance, empathy, collaboration, evocation, and support of autonomy). It has five performance factors, where each is also scored on a 7-point scale ranging from 1 - “Low ability” to 7- “High ability”. In addition, examples of “Low ability” and “High ability” (e.g., communicating with acceptance and respect, being warm and supportive) are displayed on the scoring sheet to facilitate the raters' decision-making. An overall percentage score (ranging from 14.3 to 100) is obtained for this section as follows:

$$\frac{\sum \mathrm{scores}\;\mathrm{in}\;\mathrm{each}\;\mathrm{of}\;\mathrm{the}\;\;5\;\mathrm{subsections}}{35}\times \;100$$

(3) *Behavioral Counts/MISC*: Refers to behavioural counts consistent with the MISC (e.g., affirmation, reflection, reframing, advice with permission) vs. those inconsistent (e.g., confrontation, warning). This section is scored as follows (ranging from 0 to 100):

$$\frac{\sum \mathrm{consistent}\;\mathrm{MISC}\;\mathrm{responses}\;}{\sum \mathrm{consistent}\;\&\;\mathrm{inconsistent}\;\;\mathrm{MISC}\;\mathrm{responses}\;}\times \;100$$

(4) *Global Coach Ratings – Solution Focused Interview Skills*: Refers to use of solution-focused interview skills (e.g., tolerating silences, complimenting, and amplifying solution talk). This section contains 7 items, each scored from 1- “Low ability” to 7- “High ability”. An overall percentage score (ranging from 14.2 to 100) is obtained for this section as follows:

$$\frac{\sum \mathrm{scores}\;\mathrm{in}\;\mathrm{each}\;\mathrm{of}\;\mathrm{the}\;\;6\;\mathrm{of}\;\;7\;\mathrm{rated}\;\;\mathrm{subsections}\;}{42\;\mathrm{or}\;\;49}\times \;100$$

To obtain the overall fidelity rating, an average of the four sections’ scores was computed.

Results were inputted into the Microsoft Excel Software and transferred to the IBM SPSS Statistics 27 for further statistical analyses. Descriptive statistics (means, SD, counts/proportions, ranges) were used to outline coaches' individual results per CO-FIDEL's section. Given the small sample size and lack of normal distribution of data, coaches' individual score changes over time in each of the CO-FIDEL's sections and the overall fidelity, as well as the differences between coaches' overall fidelity ratings, were examined using non-parametric tests, the *Wilcoxon Signed Rank Test* and the *Mann-Whitney U Test*, respectively. Significance was accepted at *p* < 0.05.

#### 2.6.2. Objective 2

The four-point Likert Scale responses of the questionnaire were expressed as frequencies in each category: “Not at all”, “Slightly,” “Moderately,” and “Very Much.” Responses to the open-ended questions were analyzed using a directed content-based analysis technique (26) in the NVivo Qualitative Data Analysis Software (QSR International). As a first step, the first author (TO) became familiar with all the responses to the open-ended questions. Initial codes were generated for all meaningful ideas emerging from the responses. Codes were grouped into patterns and categorized into themes and subthemes. After that, another rater reviewed the identified themes and subthemes for completeness and accuracy. This allowed for a collaborative coding and debriefing about the supported themes, enhancing the credibility and trustworthiness of the results. Any emerging discrepancies between the two coders were resolved through discussion with a third party.

## 3. Results

Between August 23^rd^, 2018, and April 3^rd^, 2019, coaches A, B, C, and D completed the entire *BRIGHT Coaching* program with eleven (*n* = 11) parent-participants. Coaches A, B, C, and D completed the program with 2, 3, 2, and 4 parent-participants, respectively. In total, 152 coaching sessions were delivered and 139 CO-FIDELs were issued. Thirteen coaching sessions (8.5%) were not assessed using CO-FIDEL secondary to audio recording and/or audio saving malfunctions.

Parent-participants (*n* = 11; 10 females – mothers; 1 male – father) were caregivers of children aged 3.7 ± 1.4 years old (8 boys; 3 girls). Some of these children were waiting to receive a diagnosis (*n* = 6), while others were waiting for services and were diagnosed with autism spectrum disorder (*n* = 5), global developmental delay (*n* = 1), intellectual disability (*n* = 1), learning disability (*n* = 1), Cohen's syndrome (*n* = 1), and speech delay (*n* = 1). On average, parent-participants were aged 37.2 ± 4.6 years old and had various educational levels ranging from master's degree (*n* = 2), vocational training (*n* = 5), college credits (*n* = 2), high school diploma (*n* = 1), to eighth grade or less (*n* = 1). Parent-participants' ethnic backgrounds included European ancestry (e.g., British, French, Irish, Italian, German) (*n* = 8), North American Aboriginal origins (e.g., First Nations, Inuit, Metis) (*n* = 2), East and South Asian (e.g., Chinese, Indian, Pakistani) (*n* = 1), and Central Asian and Middle Eastern origins (e.g., Afghan, Iranian, Lebanese) (*n* = 1). Some worked full-time (*n* = 4), while others were working part-time (*n* = 3), were stay-at-home caregivers (*n* = 2), or were temporarily on a leave (e.g., maternity, sick leave, *n* = 2). The gross family income of parent-participants ranged between $100,000–$199,999 (*n* = 4), $75,000–$99,999 (*n* = 3), $50,000–$74,999 (*n* = 2), and $35,000–$49,999 (*n* = 1).

### 3.1. Coaching fidelity

Coach A, B, and C required 3, 2, and 11 sessions respectively, to achieve and maintain a fidelity rating of ≥85% in all sections of the CO-FIDEL. Coach D achieved and maintained this threshold in all the CO-FIDEL's sections from the first coaching session.

Table 1 outlines the CO-FIDEL's results for each coach and their respective parent-participants. The overall fidelity ranged from 88.0 ± 6.3% (coach C, parent-participant_C1) to 99.5 ± 0.8% (coach D, parent-participant_D2). For intra-fidelity scores (i.e., within-coach), significant improvements were noted for coaches B and C. Specifically, coach B demonstrated a significant improvement in Section 1 of the CO-FIDEL between participant_B1 and B3 (89.9 ± 4.6 vs. 98.5 ± 2.6, *Z* = −2.74; *p* = 0.00596). Coach C showed a significant improvement in Section 4 of the CO-FIDEL between the parent-participant_C1 and C2 (82.4 ± 7.5 vs. 89.1 ± 4.1, *Z* = −2.66; *p* = 0.00758), and in the overall fidelity between parent-participant_C1 and C2 (88.67 ± 6.32 vs. 94.53 ± 1.23, *Z* = −2.66; *p* = 0. 00758). For coach A, a significant deterioration was noted for Section 1 of the CO-FIDEL between the parent-participant_A1 and A2 (99.2 ± 1.5 vs. 93.2 ± 3.4, *Z* = −2.7; *p* = 0.00694). However, no significant changes in overall fidelity scores were found. For inter-fidelity scores (i.e., between-coaches), a significant difference in the overall fidelity rating of the CO-FIDEL was found between coaches C and D for their respective parent-participants_C1 and D1, where coach D demonstrated significantly higher scores than coach C (99.2 ± 1.1 vs. 88.6 ± 6.3, Z = −3.99, *p* = 0.00006). No other significant between-coaches differences were detected.

**Table 1:** CO-FIDEL results per coach and their respective parent-participants.

**COACH A**					
	*BRIGHT Coaching* program delivered in its entity to:	Parent-participant A1	Parent-participant A2		
	*BRIGHT Coaching* sessions completed (n)	14	13		
	*BRIGHT Coaching* sessions scored with the CO-FIDEL (n)	14	13		
CO-FIDEL	SECTION 1 (%, mean ± SD)	99.20 ± 1.50	93.27 ± 3.41^a^		
	SECTION 2 (%, mean ± SD)	97.07 ± 1.69	95.58 ± 3.23		
	SECTION 3 (%, mean ± SD)	94.21 ± 9.75	99.17 ± 2.89		
	SECTION 4 (%, mean ± SD)	89.73 ± 5.80	88.37 ± 4.24		
** **	**Overall fidelity (%, mean ± SD)**	**94.94 ± 3.42**	**94.31 ± 1.95**		
**COACH B**					
	*BRIGHT Coaching* program delivered in its entity to:	Parent-participant B1	Parent-participant B2	Parent-participant B3	
	*BRIGHT Coaching* sessions completed (n)	14	13	14	
	*BRIGHT Coaching* sessions scored with the CO-FIDEL (n)	12	13	13	
CO-FIDEL	SECTION 1 (%, mean ± SD)	89.97 ± 4.61	93.89 ± 4.57	98.57 ± 2.61^a^	
	SECTION 2 (%, mean ± SD)	98.27 ± 4.15	99.22 ± 1.85	96.92 ± 4.59	
	SECTION 3 (%, mean ± SD)	96.75 ± 7.59	98.48 ± 5.03	98.46 ± 5.55	
	SECTION 4 (%, mean ± SD)	95.83 ± 6.89	94.93 ± 7.57	97.36 ± 3.36	
	**Overall fidelity (%, mean ± SD)**	**95.21 ± 4.31**	**96.63 ± 3.08**	**97.83 ± 3.29**	
**COACH C**					
	*BRIGHT Coaching* program delivered in its entity to:	Parent-participant C1	Parent-participant C2		
	*BRIGHT Coaching* sessions completed (n)	14	14		
	*BRIGHT Coaching* sessions scored with the CO-FIDEL (n)	13	9		
CO-FIDEL	SECTION 1 (%, mean ± SD)	92.34 ± 4.15	94.12 ± 2.31		
	SECTION 2 (%, mean ± SD)	90.87 ± 7.46	94.92 ± 2.38		
	SECTION 3 (%, mean ± SD)	88.74 ± 16.00	100 ± 0		
	SECTION 4 (%, mean ± SD)	82.45 ± 7.55	89.10 ± 4.13^a^		
	**Overall fidelity (%, mean ± SD)**	**88.67 ± 6.32**	**94.53 ± 1.23** ^a^		
**COACH D**					
	*BRIGHT Coaching* program delivered in its entity to:	Parent-participant D1	Parent-participant D2	Parent-participant D3	Parent-participant D4
	*BRIGHT Coaching* sessions completed (n)	14	14	14	14
	*BRIGHT Coaching* sessions scored with the CO-FIDEL (n)	11	13	14	14
CO-FIDEL	SECTION 1 (%, mean ± SD)	99.86 ± 0.48	100 ± 0	100 ± 0	99.90 ± 0.38
	SECTION 2 (%, mean ± SD)	99.14 ± 2.71	99.78 ± 0.79	99.59 ± 1.04	99.80 ± 0.76
	SECTION 3 (%, mean ± SD)	100 ± 0	100 ± 0	100 ± 0	100 ± 0
	SECTION 4 (%, mean ± SD)	97.94 ± 2.57	98.44 ± 2.72	97.96 ± 2.78	98.13 ± 2.32
	**Overall fidelity (%, mean ± SD)**	**99.22 ± 1.17**	**99.56 ± 0.84**	**99.39 ± 0.88**	**99.45 ± 0.68**

^a^ Refers to significant change in scores in comparison to Parent-participant_1 (*p* < 0.05). Note: results are presented for parent-participants and their respective coaches (A, B, C, D). For each CO-FIDEL section, results represent averages across all coaching sessions. When less than 14 sessions appear in “number of sessions completed”, this signifies that the coach delivered two topics in one session. Section 1: Coach Delivering Content & Attitude; Section 2: Global Coach Ratings/Motivational Interviewing Skills Code (MISC); Section 3: Behavioral Counts/MISC; Section 4: Global Coach Ratings – Solution Focused Interview Skills.

**Figure 1:**
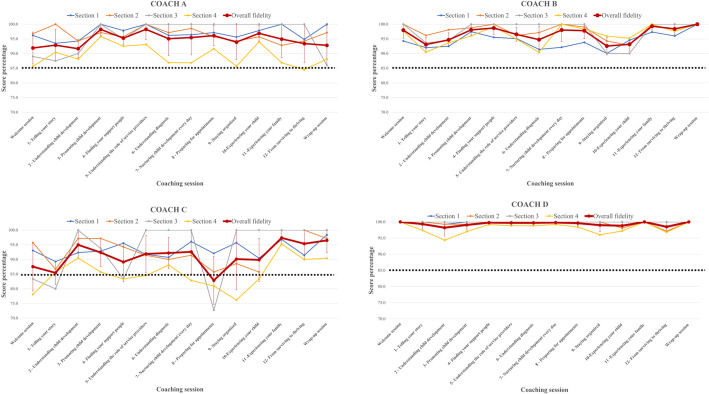
CO-FIDEL results per BRIGHT Coaching session.

Table 2 and Figures 1A–D outline the average CO-FIDEL results per *BRIGHT Coaching* session for each coach. We note an overall high and consistent coaching fidelity across coaching topics, ranging from 92.7 ± 6.03 (*Topic 1 – Telling your Story*) to 97.9 ± 3.78 (*Topic 11 – Experience your family*). Nonetheless, a higher variability was found for Section 4 of the CO-FIDEL (*Solution Focused Interview Skills (SFIS)*), as demonstrated by larger standard deviations and lower average coaching fidelity scores across all sessions for two coaches (e.g., coach A: 89.3 ± 3.6; coach C: 85.3 ± 5.2). Coaches’ B and D scores were always above 85% across all coaching topics. Coach A had one instance of ≤85.0% score (Section 4 *- SFIS*, *Topic 12- From Surviving to Thriving*, 84.5 ± 8.4%). However, this did not affect their overall fidelity score for this topic (i.e., 93.4 ± 6.5). However, coach C presented with more difficulty as four (*n* = 4) ≤ 85.0% scores were found for Section 3 (*Behavioral counts – MISC*, range: 72.7–83.4 ± 23.5), and five (*n* = 5) ≤ 85.0% scores were found for Section 4 (*SFIS*, range: 76.2–84.5 ± 1.7). In addition, coach C had a ≤85.0% overall fidelity score for *Topic 8 – Preparing for appointments* (82.9 ± 8.2), which was mainly influenced by lower scores on Sections 3 (*MISC*, 72.7%) and 4 (*SFIS,* 81.0%) of that session.

**Table 2:** CO-FIDEL results per *BRIGHT coaching* session per coach.

	Welcome session	1- Telling your story	2 - Understanding child development	3- Promoting child development	4- Finding your support people	5- Understanding the role of service providers	6- Understanding diagnosis	7- Nurturing child development every day	8 - Preparing for appointments	9- Staying organized	10 - Experiencing your child	11 - Experiencing your family	12 - From surviving to thriving	Wrap-up session	Average coaching fidelity per section mean ± SD
**COACH A (for parent-participants A1 and A2)**															** **
SECTION 1 (%, mean ± SD)	96.2 ± 1.4	93.5 ± 7.0	94.3 ± 8.1	100.0	97.9 ± 3.0	100.0 ± 0.0	96.2 ± 5.4	96.4 ± 5.1	97.1 ± 4.0	95.6 ± 3.9	97.9 ± 3.0	100.0	94.8 ± 2.8	100.0	**97.1 ± 2.2**
SECTION 2 (%, mean ± SD)	96.8 ± 0.5	100.0 ± 0.0	94.3	97.1	95.7 ± 2.0	100.0 ± 0.0	97.1	98.6 ± 2.0	95.7 ± 2.0	94.3 ± 4.0	95.7 ± 2.0	92.8 ± 2.0	94.2 ± 0.1	97.1	**96.4 ± 2.2**
SECTION 3 (%, mean ± SD)	89.0 ± 15.6	87.5 ± 17.7	90.0 ± 14.1	100.0	95.0 ± 7.1	100.0 ± 0.0	100.0 ± 0.0	100.0 ± 0.0	100.0 ± 0.0	100.0 ± 0.0	100.0 ± 0.0	100.0 ± 0.0	100.0 ± 0.0	86.0	**96.3 ± 5.6**
SECTION 4 (%, mean ± SD)	85.7 ± 10.1	90.5 ± 6.7	88.1 ± 6.7	95.8	92.5 ± 3.8	93.1 ± 4.7	86.9 ± 5.0	86.9 ± 1.6	91.6 ± 1.6	85.7 ± 0.0	94.0 ± 5.1	86.9 ± 1.7	84.5 ± 8.4	88.1	**89.3 ± 3.6**
**Overall fidelity (%, mean ± SD)**	**91.9 ± 5.5**	**92.9 ± 5.4**	**91.7 ± 3.1**	**98.2 ± 2.1**	**95.3 ± 2.2**	**98.3 ± 3.4**	**95.1 ± 5.7**	**95.5 ± 5.9**	**96.1 ± 3.5**	**93.9 ± 6.0**	**96.9 ± 2.6**	**94.9 ± 6.3**	**93.4 ± 6.5**	**92.8 ± 6.9**	** **
**COACH B (for parent-participants B1, B2, B3)**															
SECTION 1 (%, mean ± SD)	94.3 ± 7.6	92.0 ± 7.0	92.5 ± 6.5	97.4 ± 2.4	95.5 ± 4.3	95.1 ± 3.2	91.4 ± 7.6	92.1 ± 7.1	93.8 ± 7.3	90.1 ± 8.0	94.6 ± 3.6	97.3 ± 4.6	96.0 ± 5.7	100.0	**94.4 ± 2.7**
SECTION 2 (%, mean ± SD)	100.0 ± 0.0	96.2 ± 4.4	98.1 ± 3.3	98.8 ± 2.1	100.0 ± 0.0	96.2 ± 6.6	97.1 ± 2.9	100.0 ± 0.0	99.0 ± 1.7	94.3 ± 8.1	92.9 ± 10.1	100.0 ± 0.0	100.0 ± 0.0	100.0	**98.0 ± 2.4**
SECTION 3 (%, mean ± SD)	100.0 ± 0.0	93.7 ± 11.0	94.4 ± 9.6	100.0 ± 0.0	100.0 ± 0.0	100.0 ± 0.0	100.0 ± 0.0	100.0 ± 0.0	100.0 ± 0.0	90.0 ± 14.1	90.0 ± 14.1	100.0 ± 0.0	100.0 ± 0.0	100.0	**97.7 ± 3.9**
SECTION 4 (%, mean ± SD)	97.6 ± 4.1	90.5 ± 12.6	93.6 ± 3.6	96.0 ± 6.9	99.2 ± 1.4	94.9 ± 4.8	90.5 ± 12.6	100.0 ± 0.0	98.4 ± 2.8	95.9 ± 2.4	95.2 ± 6.7	100.0 ± 0.0	97.6 ± 0.0	100.0	**96.4 ± 3.2**
**Overall fidelity (%, mean ± SD)**	**98.0 ± 2.7**	**93.1 ± 2.4**	**94.7 ± 2.4**	**98.1 ± 1.7**	**98.7 ± 2.1**	**96.6 ± 2.4**	**94.8 ± 4.6**	**98.0 ± 3.9**	**97.8 ± 2.8**	**92.6 ± 3.0**	**93.2 ± 2.3**	**99.3 ± 1.3**	**98.4 ± 2.0**	**100.0 ± 0.0**	** **
**COACH C (for parent-participants C1 and C2)**															** **
SECTION 1 (%, mean ± SD)	93.1 ± 3.8	89.3 ± 5.1	92.3 ± 2.7	92.9	95.6 ± 1.8	91.7 ± 0.4	90.7 ± 7.1	96.1 ± 3.5	92.1	95.7	90.4	96.8	91.4	98.4	**93.3 ± 2.7**
SECTION 2 (%, mean ± SD)	95.7 ± 6.1	86.7 ± 14.8	97.1 ± 4.0	97.1 ± 4.0	94.3 ± 0.0	91.4 ± 4.1	90.0 ± 6.1	91.4 ± 4.0	85.7	88.6	85.7	-	100.0	97.1	**92.4 ± 4.9**
SECTION 3 (%, mean ± SD)	83.4 ± 23.5	80.0 ± 28.3	100.0 ± 0.0	93.8 ± 8.8	83.3 ± 23.6	100.0 ± 0.0	100.0 ± 0.0	100.0 ± 0.0	72.7	100.0	100.0	100.0	100.0	100.0	**93.8 ± 9.6**
SECTION 4 (%, mean ± SD)	78.1 ± 17.5	85.7 ± 6.7	90.5 ± 3.4	85.7 ± 6.7	83.3 ± 0.0	84.5 ± 1.7	88.1 ± 13.5	82.9 ± 4.0	81.0	76.2	83.3	95.2	90.0	90.4	**85.3 ± 5.2**
**Overall fidelity (%, mean ± SD)**	**87.6 ± 8.2**	**85.4 ± 3.9**	**95.0 ± 4.4**	**92.4 ± 4.8**	**89.1 ± 6.7**	**91.9 ± 6.3**	**92.2 ± 5.3**	**92.6 ± 7.4**	**82.9 ± 8.2**	**90.1 ± 10.4**	**89.9 ± 7.4**	**97.4 ± 2.4**	**95.4 ± 5.4**	**96.5 ± 4.2**	** **
**COACH D (for parent-participants D1, D2, D3, and D4)**															** **
SECTION 1 (%, mean ± SD)	100.0 ± 0.0	100.0 ± 0.0	99.2 ± 0.9	100.0 ± 0.0	100.0 ± 1.4	100.0 ± 0.0	100.0 ± 0.0	100.0 ± 0.0	100.0 ± 0.0	100.0 ± 0.0	100.0 ± 0.0	100.0 ± 0.0	100.0 ± 0.0	100.0 ± 0.0	**99.9 ± 0.2**
SECTION 2 (%, mean ± SD)	100.0 ± 0.0	100.0 ± 0.0	99.3 ± 1.4	99.3 ± 1.4	100.0 ± 0.0	100.0 ± 0.0	100.0 ± 0.0	100.0 ± 0.0	100.0 ± 0.0	100.0 ± 0.0	98.1 ± 1.7	100.0 ± 0.0	97.1 ± 5.0	100.0 ± 0.0	**99.6 ± 0.9**
SECTION 3 (%, mean ± SD)	100.0 ± 0.0	100.0 ± 0.0	100.0 ± 0.0	100.0 ± 0.0	100.0 ± 0.0	100.0 ± 0.0	100.0 ± 0.0	100.0 ± 0.0	100.0 ± 0.0	100.0 ± 0.0	100.0 ± 0.0	100.0 ± 0.0	100.0 ± 0.0	100.0 ± 0.0	**100.0 ± 0.0**
SECTION 4 (%, mean ± SD)	100.0 ± 0.0	97.4 ± 0.2	94.4 ± 3.8	97.0 ± 3.6	99.2 ± 1.7	98.8 ± 2.4	98.8 ± 2.4	99.2 ± 1.4	98.4 ± 1.4	96.0 ± 1.4	97.2 ± 2.1	100.0 ± 0.0	96.8 ± 3.6	100.0 ± 0.0	**98.1 ± 1.7**
**Overall fidelity (%, mean ± SD)**	**100.0 ± 0.0**	**99.3 ± 1.3**	**98.2 ± 2.6**	**99.1 ± 1.4**	**99.8 ± 0.4**	**99.7 ± 0.6**	**99.7 ± 0.6**	**99.8 ± 0.4**	**99.6 ± 0.8**	**99.0 ± 2.0**	**98.8 ± 1.4**	**100.0 ± 0.0**	**98.5 ± 1.7**	**100.0 ± 0.0**	** **
**Average coaching fidelity per session (%, mean ± SD)**	**94.4 ± 6.85**	**92.7 ± 6.03**	**94.9 ± 3.75**	**96.9 ± 3.75**	**95.7 ± 5.42**	**96.6 ± 4.56**	**95.4 ± 4.91**	**96.5 ± 5.38**	**94.1 ± 8.0**	**93.9 ± 6.53**	**94.7 ± 5.14**	**97.9 ± 3.78**	**96.4 ± 4.52**	**97.3 ± 4.72**	

Results are presented as averages across parent-participants and their respective coaches (A, B, C, D). Section 1: Coach Delivering Content & Attitude; Section 2: Global Coach Ratings/Motivational Interviewing Skills Code (MISC); Section 3: Behavioral Counts/MISC; Section 4: Global Coach Ratings – Solution Focused Interview Skills. Sections in red represent those where the coaching fidelity was less than 85%. Sections without SD represent those where there was only one value (e.g., missing audio recording from a session to compute an average).

**Table:** 

25%	
50%	
75%	
100%	

### 3.2. Coaches' satisfaction with and usefulness of the tool

Table 3 outlines coaches' responses to the Likert Scale statements about satisfaction with the CO-FIDEL and its usefulness. Three to four coaches (75%–100%) report moderate to high satisfaction with CO-FIDEL's content, format, administration frequency, understandability, comprehensiveness, relevance to the training needs as a coach, and usefulness/helpfulness to the coach. Three to four (75%–100%) coaches indicated feeling “moderately” to “very much” assured/positive/motivated when reviewing their CO-FIDEL results; understood/supported by the Lead Coach when reviewing their ratings; and that they have benefited from the use of the CO-FIDEL.

**Table 3:** Coaches responses: satisfaction and usefulness of CO-FIDEL.

	Not at all	Slightly	Moderately	Very much
**Extent to which coaches are satisfied with the CO-FIDEL's:**				
Content				
Format				
Administration frequency understandability				
understandability				
Comprehensiveness (complete/covers all important items)				
Relevance to the coaches’ training needs				
Usefulness/helpfulness to the coach				
**Extent to which do coaches:**				
Feel assured/positive/motivated when reviewing their CO-FIDEL				
Feel understood/supported by the lead coach when reviewing their CO-FIDEL				
Feel that they benefit from the use of the CO-FIDEL				

Responses to the open-ended questions revealed two main themes (Table 4): *Facilitators or positive impacts* (skill improvement, maintenance, and highlighting progress; comprehensiveness of the tool; validation of skill alignment with the intervention) and *Barriers* or *areas of improvement with respect to the CO-FIDEL* (applicability to all sessions; ceiling effect; snapshot of the performance; missing elements). Coaches reported more positive impacts related to the use of the CO-FIDEL than challenges (i.e., 7 vs. 4 statements). Coaches conveyed that the main benefit of the CO-FIDEL use was that it allowed them to pinpoint specific areas for improvement and highlight progress. For instance, one coach stated: “*The CO-FIDEL helps me to notice trends where I could improve and also highlights my progress. It is a great tool to prompt self-reflection.*”

**Table 4:** Statement examples for emerging sub-themes of facilitators & barriers.

	Subtheme Number of statements (n)	Salient quotes
**FACILITATORS**	Skill improvement, maintenance, and highlighting progress (*n* = 5)	“The CO-FIDEL helps me to notice trends where I could improve and also highlights my progress. It is a great tool to prompt self-reflection.” “Helped me understand what I needed to change.” “When you are in the middle of the session, or even afterwards, it is hard to get an accurate perception and self-evaluation of the work you’ve done, so having CO-FIDEL form completed is a nice guidepost to have as part of the clinical supervision.”
	Comprehensiveness of the tool (*n* = 1)	“I like how comprehensive the CO-FIDEL is and that it includes information pertaining to our delivery of the manual content as well as our interactions with the participant. I especially appreciate the general comments.”
	Validation of skill alignment with the intervention (*n* = 1)	“I think it provides a good summary of the necessary skills and approach desired by the program. It has been useful to know that my work is in line with the chosen strategies of the program. We work in an isolated way […], I do sometimes wonder if my work is what the research team is hoping it would be. Getting feedback through the CO-FIDEL helps with that.”
**BARRIERS**	Applicability to all sessions (*n* = 1)	“[…] sometimes the particular topic you are covering is more “educational or technical” and therefore the coach could score low on things like empathy or evocation […] that are more present in topics that have more reflections and are more emotions-based.”
	Ceiling effect (n=1)	“My scores are usually quite good, but there is, of course, still room for improvement. However, the improvements that I want or need to make don’t always come across on the form.”
	Snapshot (*n* = 1)	“[The CO-FIDEL] gave me a snap-shot of how I am doing but not more than that, I think a more detailed comprehensive evaluation as well as maybe a [broader] one every six months with specific goals to work on could be helpful in terms of the coach's evolution.”
	Missing elements (*n* = 1)	“I wish the CO-FIDEL could somehow capture the overall alliance with the participant. I take the setting events and general vibe of a session in consideration when working through the material. For example, if the participant has been up all night with their sick child and is exhausted, I may not push, probe or ask additional/spontaneous questions. I think the coaching alliance and the participant's overall engagement/enjoyment of the session is incredibly important.”

## 4. Discussion

We presented a novel rating tool developed by our team to evaluate the fidelity of coaches in delivering a health coaching intervention to parents of children with or with suspected developmental delays, called CO-FIDEL (**CO**aches **F**idelity in **I**ntervention **DEL**ivery). In addition to outlining the development procedures of this tool, we sought to demonstrate CO-FIDEL's feasibility in evaluating coaches' fidelity and its change over time during the pilot phase of the *BRIGHT Coaching* randomized clinical trial; and to appreciate coaches' perspectives in the usefulness and satisfaction with the tool. To our knowledge, this is the first study to examine and present in detail the process of fidelity evaluation of coaches in delivering an online health coaching program in the context of childhood disability.

We showed that it is feasible to use the CO-FIDEL to evaluate the fidelity of coaching skills. Our study determined that the overall coaching fidelity was higher than the 85% threshold for all coaches in their first round of providing the intervention. It remained high for subsequent participants of each coach, and for one coach, it improved considerably from the first to the second participant. Similarly, two coaches showed significant improvements over time in certain sections of the CO-FIDEL. We propose that the iterative nature of the training (i.e., assessment following each coaching session provided to coaches as feedback) and the *a priori* extensive training provided to the coaches are the basis of high initial overall fidelity scores and its maintenance. In fact, coaches took on average four sessions to achieve and maintain a ≥85% fidelity score in every section of the CO-FIDEL. Overall, coaches demonstrated consistently high overall fidelity scores across the different *BRIGHT Coaching* topics. However, two of the coaches obtained somewhat lower scores on Sections 3 (*MISC*) and 4 (*SFIS*) of the CO-FIDEL for certain topics. This was the case for *Topic 12 – From Surviving to Thriving* and *Topic 8 – Preparing for appointments*. Sections 3 and 4 of the CO-FIDEL refer to the coach's ability and behavioural counts in using motivational and solution-focused interviewing skills (e.g., open-ended questions, summarize, tolerate and use silences, and compliment and affirm client's perceptions). We speculate that the inherent challenges of the coaching topics may be at play and the novelty of the coaches to the program. More specifically, for *Topic 12 – From Surviving to Thriving*, the subject discussed can be difficult and sensitive for caregivers in times of uncertainty and vulnerability (e.g., still waiting for a diagnosis). For coaches, it could be a challenging session to evoke SFIS such as complimenting or amplifying solution talk, as well as being able to easily balance between manual content and spontaneous counselling. Similarly, for *Topic 8 – Preparing for appointments*, a coach may tend to use more inconsistent responses such as advice or direct without permission given that the content covered in this session is more didactic in nature. In addition, it should be considered that the coaches were new to the *BRIGHT Coaching* program and the parent-participants were their first participants. Nonetheless, is encouraging to observe that one of the coaches significantly improved their scores in Section 4 of the CO-FIDEL with their second parent-participant, suggesting that iterative feedback provided *via* the CO-FIDEL may support coaching skill progression.

In gathering coaches' perspectives on the usefulness of and the satisfaction with the CO-FIDEL, it emerged that they highly appreciated this tool in validating and improving their skills as they delivered the *BRIGHT Coaching* program. Nonetheless, reported barriers among others such as the possible ceiling effect, snapshot feature without follow-up/continuity across sessions, and missing elements. In response to those challenges, our team proposes the following solutions. For the possible ceiling effect, where a coach is scored high by the assessor but feels that improvements are still needed, we propose that the CO-FIDEL could be filled out by the coach him/herself and compared in a discussion session with the assessor to evaluate areas of discrepancies. In addition, we could increase the scale (currently out of 7, ranging from 1 to 7) to range from 0 to 10, similarly to a Visual Analogue Scale, which potentially leaves more room for interpretation and scoring. For the lack of continuity across sessions (i.e., the snapshot nature of the CO-FIDEL), we propose that scores could be inputted into prepared tables right after the scoring is completed, generating an instant graphical representation of the results. Those could be provided to the coach for reference, where their progress is visible from one session to the next. In addition, CO-FIDEL could be applied to randomly selected sessions coaching sessions post-pilot (e.g., in a trial or in an implemented program), to ensure a coach maintains their skills and abilities in providing the intervention. For missing elements, we propose to add items relating to coaching alliance and participant level of enjoyment and engagement in the session into Section 1 of the tool.

Coaching in childhood disability can be parent- and/or child-targeted, depending on the focus of the program and the intended outcomes (i.e., child and/or parent-related outcomes) (6). *BRIGHT Coaching* is designed to support and empower caregivers of children with suspected developmental challenges who are waiting for services. In addition, it addresses child-related needs in three coaching topics (e.g., *Topic 3 and 4 - Understanding/nurturing child development*, *Topic 7 - Nurturing child development everyday*). Therefore, it is a parent and a child-focused program (i.e., mixed coaching approach). Similarly, the Occupational Performance Coaching (OPC) (27), a program with a standardized fidelity measure developed in 2020 (i.e., after the *BRIGHT Coaching* randomized clinical trial pilot phase) (28), employs a mixed coaching approach. The focus of the OPC is on achieving the child's occupational performance goals. In addition, it was found to optimize parent-related outcomes such as parental competence/self-efficacy and knowledge/insight. The OPC's fidelity has been recently examined (29). The use of the mixed coaching approach is also reflected in the used OPC Fidelity Measure, which has items specific to both children and caregivers. Moreover, the OPC Fidelity measure incorporates the assessment of the use of motivational and solution-focused interviewing skills (e.g., *Item 1- Therapist expressed empathy through comment & gesture, comprising non-judgemental responsiveness to the client's emotional experience*). Therefore, the CO-FIDEL emerges as one of the several available options for measuring the fidelity of delivering a mixed coaching approach program, where motivational and solution-focused interviewing skills are employed by the coach.

We recognize the limitations of this study. The main shortcoming is that only the Lead Coach administered the CO-FIDEL. This was also the person who trained and continued to supervise and support the coaches, and therefore, might have been biased. Psychometric properties were not measured in the scope of this study. We also have a small sample size of coaches and pilot participants. We experienced some malfunction of the audio recording equipment/process of the coaching sessions (*n* = 13/152), leading to the loss of data needed to complete the CO-FIDEL for these sessions. However, this is a tolerable extent of missing data, given that 139 sessions were fully and successfully evaluated. Following the pilot phase of the trial, we made a switch to a new online meeting platform, accepting smaller bandwidth, which has shown to be working properly.

To further optimize the validity of the coaching skills during the *BRIGHT Coaching* trial recruitment phase of the first two to three parent-participants, the CO-FIDEL was applied by the Lead Coach on randomly selected sessions for every coach, as follows: Fidelity Check 1 - randomly picked session from the first half of the program (Welcome session to Topic 6); Fidelity Check 2- randomly picked session from the second half of the program (Topic 7 to Wrap-up). If the coach reported that they had challenges or needed more guidance and feedback about a particular session, this session is assessed in addition to the randomly picked session. The Lead Coach also has maintained team and individual meetings to allow coaches to report and discuss difficulties and celebrate successes and improvements.

In conclusion, we aim to emphasize the importance of assessing coaching fidelity, as this plays an important role interpreting the results of an intervention study in terms of the validity of the findings. Its use highlighted coaches' strengths, weaknesses, skills improvement, and maintenance. We strongly encourage future teams conducting projects in health coaching to evaluate and describe processes related to intra- and inter-coach fidelity. In future research, we will focus on fortifying the CO-FIDEL according to the solutions proposed and study its psychometric properties.

## Data Availability

The raw data supporting the conclusions of this article will be made available by the authors, without undue reservation.
