# Supplementary material for: Pediatric tele-coaching fidelity evaluation: Feasibility, perceived satisfaction and usefulness of a new measure
Source: Front Rehabil Sci. 2023 Feb 21;4:1057641. doi: 10.3389/fresc.2023.1057641 (PMC9989194; doi:10.3389/fresc.2023.1057641)
Supplement: Supplementary file 1 [file Image1.pdf]

Supplementary Material 1 Overview of the *BRIGHT Coaching* program

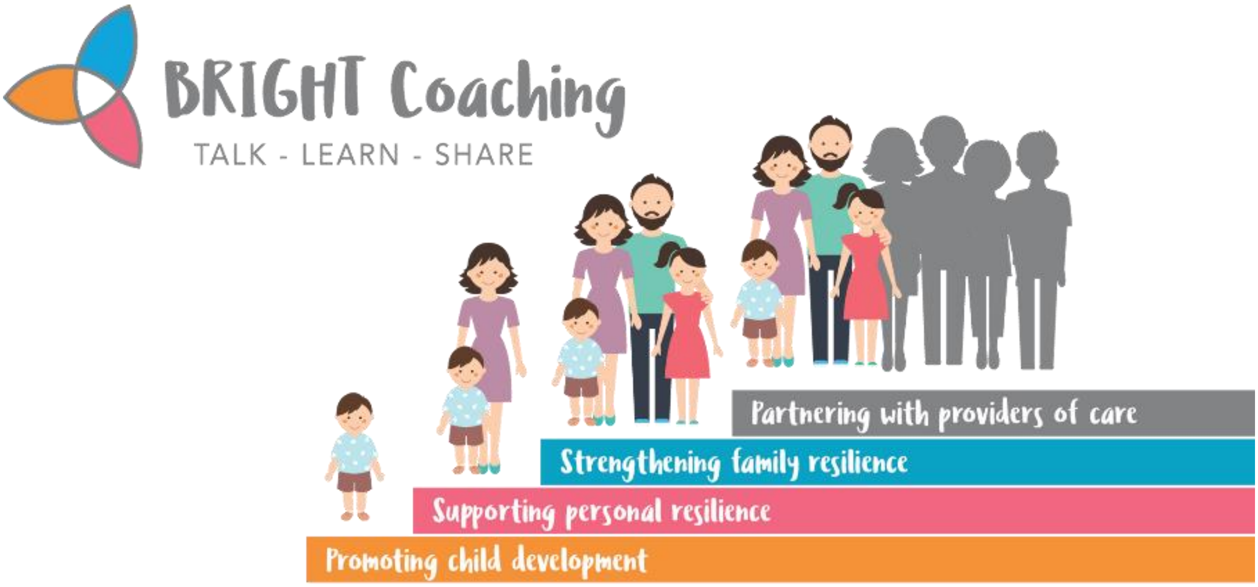

**Welcome to BRIGHT Coaching**
**Topic 1: Telling your story**
**Topic 2: Understanding child development**
**Topic 3: Supporting child development**
**Topic 4: Finding your support people**
**Topic 5: Understanding the roles of service providers**
**Topic 6: Understanding diagnosis**
**Topic 7: Nurturing child development every day**
**Topic 8: Preparing for appointments**
**Topic 9: Staying organized**
**Topic 10: Experiencing your child**
**Topic 11: Experiencing your family**
**Topic 12: From surviving to thriving**
**Wrap-up**

TALK
TO A COACH THROUGH THIS TRANSITION

LEARN
HOW YOU CAN SUPPORT YOUR CHILD

SHARE
AND CONNECT WITH OTHER PARENTS
