# Supplementary material for: Pediatric tele-coaching fidelity evaluation: Feasibility, perceived satisfaction and usefulness of a new measure
Source: Front Rehabil Sci. 2023 Feb 21;4:1057641. doi: 10.3389/fresc.2023.1057641 (PMC9989194; doi:10.3389/fresc.2023.1057641)
Supplement: Supplementary file 2 [file Image2.pdf]

Supplementary Material 2 Development process of the CO-FIDEL

DEVELOPMENT OF CO-FIDEL

(1) Literature review of the intervention studies related to coaching fidelity ascertainment procedures

(2) An opinion-seeking technique from experts in the field of coaching and childhood disability

CO-FIDEL's development was aligned with the *Comprehensive Intervention Fidelity Guide (Componenet 3-Monitoring Intervention Delivery) (*Gearing et al., 2011)

DEVELOPMENT PROCEDURE

1) ***BRIGHT Coaching*** published a **systematic review** and **analysis** on existing coaching interventions provided to parents of children with disability (Ogourtsova et al., 2019). Included **28 intervention studies,** extracting information on coaches training and fidelity ascertainment.

2) Four (n=4) **experts** in the field of family coaching and childhood disability participated

Review results presented to the participating experts

Discussion on an optimal coaching fidelity evaluation tool: ***Content, nature, administration method, frequency of administration, and scoring***

PROPOSED DESIGN OF CO-FIDEL:

Expert discussion determined measurement elements reflective of:
BRIGHT Coaching program (Majnemer, O'Donnell, Ogourtsova et al., 2021)
Overall childhood disability coaching principles
Fidelity measurement.

1) Types of behaviors to be measured: Behaviors that are program-specific, essential, and behaviors that need to be avoided (Waltz et al, 1993).

2) Coaching competence: The level of engagement with participant (Santacroce et al., 2004), and the sensitivity with which the treatment protocol was applied (Perepletchikova et al., 2007).

3) Measures: Frequency counts of particular behaviors (Waltz et al, 1993) and use of a rating scale to better reflect rater's true evaluation (Preston et al., 2000).

CO-FIDEL DEVELOPMENT RESULTS

Only **6 out of the 28** included studies in the systemic review referred to the ascertainment of coaches' fidelity in delivering their health coaching program to parents of children with developmental disabilities.

Systemic review and team discussion among experts in the fields of coaching and childhood disability found:

1. The fidelity assessment procedure was not discussed in enough detail.

2. The method of fidelity assessment was either a review of an audiotaped or a videotaped session or supervision of active cases by the principal investigator or an accredited practitioner.

3. Evaluation frequency ranged from weekly to bi-monthly.

4. Only one study specified that coaches needed to attain a fidelity score of >90% to begin the provision of the intervention to study participants.

****Following the review of findings and team expert discussion***

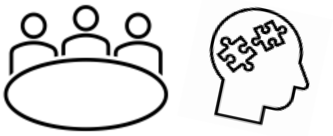

Incorperated principles of the Interview Skills Code (MISC) (Miller et al., 2003; Miller & Rose, 2009) and the Solution-Focused Interview Skills (SFIS) (Bannink, 2010; Cheng, 2007) into the fidelity rating tool

Consistent **MISC** responses included: Advise with permission, affirm, emphasize control, question openly, reflect, reframe, and support.

Non-consistent **MISC** responses included: Advise without permission, confront, direct, raise concern without permission and warn (Miller et al., 2003; Miller & Rose, 2009).

SFIS responses include: Open-ended questions, summaries, tolerating and using silences, complimenting participants' strengths and past/ current successes, and affirming client's perception (Bannink, 2010; Cheng, 2007)

The two concepts of **MISC and SFIS** influenced the development of the 4 ratable sections of of **CO-FIDEL.**

Version 1.0 of the CO-FIDEL was created.
