# Supplementary material for: Pediatric tele-coaching fidelity evaluation: Feasibility, perceived satisfaction and usefulness of a new measure
Source: Front Rehabil Sci. 2023 Feb 21;4:1057641. doi: 10.3389/fresc.2023.1057641 (PMC9989194; doi:10.3389/fresc.2023.1057641)
Supplement: Supplementary file 3 [file Image3.pdf]

Supplementary Material 3

Table 1 Training and assessment of coaches in health coaching programs for parents of children with developmental disabilities

Reference	Coaches characteristics	Coaches training	Coaches fidelity evaluation
Feinberg et al., 2014 (1)	Educators representing range of disciplines within early intervention programs (child development, nursing, speech and language, social work) and graduate students.	1 day of didactics + 3 to 5 practice sessions conducted under the supervision of the investigators;<br><br>Training sessions were videotaped, and all educators received individualized feedback until they have meet predefined criteria for protocol adherence;<br><br>Educators meet 2 times/week at each site for supervision with investigators.	**Content**: NA<br><br>**Method**: audiotaped one randomly selected training/coaching sessions from each educator and employed a standardized form to assess whether all components of intervention were present and delivered as per protocol<br><br>**Frequency**: NA
Kasari et al., 2015 (2)	Therapists were PhD candidates in Psychology.	Prior to the beginning of the coaching, therapists were required to demonstrate >90% fidelity on standardized measures designed for the intervention.<br><br>Training was delivered to them over 2-6 months (no other specifics given)	**Content**: NA<br><br>**Method**: Project coordinator used a random numbers list to select sessions for fidelity ratings.<br><br>**Frequency**: Supervision of active cases was conducted weekly.
Magana et al., 2017 (3)	“Promotoras” – mothers of children with ASD, Spanish speaking/Latin American.	48-hour training: manual content and materials were presented, discussed and role played and research training according to the ethics review board.	**Content:** NA<br><br>**Method:** Monitor and feedback by a project coordinator;

			**Frequency:** between each sessions and weekly telephone calls.
Nguyen et al., 2016 (4)	Therapists with post-graduate levels of education	Training workshop; no other details provided.	**Content:** NA<br><br>**Method:** review of cases in a group setting with the PI;<br><br>**Frequency:** weekly for 2 hours.
Whittingham et al., 2009 (5)	Probationary psychologists	2-day training workshop	**Content:** NA<br><br>**Method:** supervision sessions with an accredited practitioner;<br><br>**Frequency:** weekly.
Stahmer et al., 2017 (6)	Individuals with degree in their primary discipline (behavior specialist, SLP, special educators, family/child counselors)	2-day training workshop (16 hours total): didactic presentations, video examples, role-playing activities, small and large group discussions	**Content:** checklist, comments;<br><br>**Method:** performance feedback via video or live observation regarding the implementation. All therapists received feedback (regardless of their performance). Occurred either in-person or via technology connection (skype, videoconference, etc.)<br><br>**Frequency:** Bimonthly

Legend: Autism spectrum disorder (ASD); principal investigator (PI); not available (NA).

1. Feinberg E, Augustyn M, Fitzgerald E, Sandler J, Ferreira-Cesar Suarez Z, Chen N, et al. Improving maternal mental health after a child's diagnosis of autism spectrum disorder: results from a randomized clinical trial. JAMA Pediatr. 2014;168(1):40-6.

2. Kasari C, Gulsrud A, Paparella T, Hellemann G, Berry K. Randomized comparative efficacy study of parent-mediated interventions for toddlers with autism. J Consult Clin Psychol. 2015;83(3):554-63.
3. Magana S, Lopez K, Machalicek W. Parents Taking Action: A Psycho-Educational Intervention for Latino Parents of Children With Autism Spectrum Disorder. Fam Process. 2017;56(1):59-74.
4. Nguyen CT, Fairclough DL, Noll RB. Problem-solving skills training for mothers of children recently diagnosed with autism spectrum disorder: A pilot feasibility study. Autism. 2016;20(1):55-64.
5. Whittingham K, Sofronoff K, Sheffield J, Sanders MR. Stepping Stones Triple P: an RCT of a parenting program with parents of a child diagnosed with an autism spectrum disorder. J Abnorm Child Psychol. 2009;37(4):469-80.
6. Stahmer AC, Brookman-Frazee L, Rieth SR, Stoner JT, Feder JD, Searcy K, et al. Parent perceptions of an adapted evidence-based practice for toddlers with autism in a community setting. Autism. 2017;21(2):217-30.
