# Supplementary material for: Pediatric tele-coaching fidelity evaluation: Feasibility, perceived satisfaction and usefulness of a new measure
Source: Front Rehabil Sci. 2023 Feb 21;4:1057641. doi: 10.3389/fresc.2023.1057641 (PMC9989194; doi:10.3389/fresc.2023.1057641)
Supplement: Supplementary file 4 [file Image4.pdf]

Supplementary Material 4 CO-FIDEL Version 1.0

CO-FIDEL - COaches **F**idelity in **I**ntervention **DEL**ivery

Partly adopted from the Motivational Interviewing Skill Code 2.1 (**MISC**) and the Solution Focused Interviewing Skills (**SFIS**) & Questions

DESCRIPTORS:

**Coach:**	
**Topic:**	
**Session #:**	
**Date of session:**	
**Evaluation #:**	
**Participants on call:**	**Mother** ☐ **Father** ☐ **Grandparent** ☐ **Foster parent** ☐ **Other** ☐
**Evaluation form completed by:**	
**Evaluation form completed on:**	
**Timeframe used to complete MISC:**	

GENERAL COMMENTS:

OVERVIEW: COACH DELIVERY / CONTENT / APPROACH (out of 70)

**The extent to which the coach …**	**1- LOW**	**2**	**3**	**4**	**5**	**6**	**7- HIGH**
1. is able to guide the participants through the content of the session	☐	☐	☐	☐	☐	☐	☐
2. covers all the content for the session	☐	☐	☐	☐	☐	☐	☐
3. is knowledgeable about the activities and encourages the participants to complete them	☐	☐	☐	☐	☐	☐	☐
4. respects/follows the content	☐	☐	☐	☐	☐	☐	☐
5. respects the pace of the participant	☐	☐	☐	☐	☐	☐	☐
6. ensures the participant understands the content	☐	☐	☐	☐	☐	☐	☐
7. is interested / curious during the session	☐	☐	☐	☐	☐	☐	☐
8. respects the timeline of the session	☐	☐	☐	☐	☐	☐	☐
9. respects the boundaries with the participant	☐	☐	☐	☐	☐	☐	☐
10. is able to balance “spontaneous” counseling vs. coaching manual content delivery	☐	☐	☐	☐	☐	☐	☐

GLOBAL COACH RATINGS – MISC (out of 35)

** Definitions and terms provided in the Manual for the Motivational Interviewing Skill Code (MISC) Version 2.1 by Miller, W.R., Moyers, T.B., Ernst, D., & Amrhein, P., (2008): Center on Alcoholism, Substance Abuse and Addictions, The University of New Mexico; available online at https://casaa.unm.edu/download/misc.pdf

**ACCEPTANCE**: The extent to which the coach communicates unconditional positive regard to the client:							
**1-LOW**<br>judgemental, harsh, disrespectful, labelling, condescending**	**2**	**3**	**4**	**5**	**6**	**7-HIGH**<br>communicates<br>with acceptance, and respect, is being warm and supportive	**N/A**
☐	☐	☐	☐	☐	☐	☐	☐
**EMPATHY**: The extent to which the coach understands and/or makes an effort to accurately understand the client's perspective:							
**1-LOW**<br>shows little interest in the client's own perspective and experiences, little effort to gain a deeper understanding, lacking reflective listening **	**2**	**3**	**4**	**5**	**6**	**7-HIGH**<br>show active interest in making sure they understand the client, include client's perceptions, situations, meanings, and feelings; accurately follows client's statements to gain clarity	**N/A**
☐	☐	☐	☐	☐	☐	☐	☐
**SPIRIT**: The extent to which the coach uses motivational interviewing skills of collaboration, evocation and autonomy-supportive:							
**Collaboration:** coach negotiates with the client and avoids an authoritarian stance; shows respect for ideas, accepts differences and client's points of views.							
**1-LOW**	**2**	**3**	**4**	**5**	**6**	**7-HIGH**	**N/A**
☐	☐	☐	☐	☐	☐	☐	☐
**Evocation:** coach draws out the client's perspective rather than installing the coaches' knowledge, insight and advice; does not educate or give opinions without permission, is curious and patient.							
**1-LOW**	**2**	**3**	**4**	**5**	**6**	**7-HIGH**	**N/A**
☐	☐	☐	☐	☐	☐	☐	☐
**Autonomy-supportive:** coach emphasizes the client's freedom of choice and conveys an understanding that the critical variables for change are within the client and cannot be imposed by others.							
**1-LOW**	**2**	**3**	**4**	**5**	**6**	**7-HIGH**	**N/A**
☐	☐	☐	☐	☐	☐	☐	☐

BEHAVIOR COUNTS - MISC

Utterance/Statement	MI-CONSISTENT (MIC, +) RESPONSES							MI-INCONSISTENT (MIIN, -) RESPONSES				
	Advise with permission	Affirm	Emphasize control	Open Question	Reflect	Reframe	Support	Advise without permission	Confront	Direct	Raise concern without permission	Warn
	☐	☐	☐	☐	☐	☐	☐	☐	☐	☐	☐	☐
	☐	☐	☐	☐	☐	☐	☐	☐	☐	☐	☐	☐
	☐	☐	☐	☐	☐	☐	☐	☐	☐	☐	☐	☐
	☐	☐	☐	☐	☐	☐	☐	☐	☐	☐	☐	☐
	☐	☐	☐	☐	☐	☐	☐	☐	☐	☐	☐	☐
	☐	☐	☐	☐	☐	☐	☐	☐	☐	☐	☐	☐
	☐	☐	☐	☐	☐	☐	☐	☐	☐	☐	☐	☐

GLOBAL COACH RATINGS – SFIS (out of 42 or 49, depending if the last skill item used by coach)

1. **OPEN-ENDED QUESTIONS**: The extent to which the coach uses open-ended questions during the session.							
**1-LOW**	**2**	**3**	**4**	**5**	**6**	**7-HIGH**	**N/A**
☐	☐	☐	☐	☐	☐	☐	☐
2. **SUMMARIZING**: The extent to which the coach periodically states back to the client his/her thoughts, actions, and feelings.							
**1-LOW**	**2**	**3**	**4**	**5**	**6**	**7-HIGH**	**N/A**
☐	☒	☐	☐	☐	☐	☐	☐
3. **TOLERATING/USING SILENCE**: The extent to which the coach allows time for the participant to come up with their own responses; and avoid temptation to fill in silence with advice.							
**1-LOW**	**2**	**3**	**4**	**5**	**6**	**7-HIGH**	**N/A**
☐	☐	☐	☐	☐	☐	☐	☐
4. **COMPLIMENTING**: The extent to which the coach acknowledges the participants strengths and past successes.							
**1-LOW**	**2**	**3**	**4**	**5**	**6**	**7-HIGH**	**N/A**
☐	☐	☐	☐	☐	☐	☐	☐
5. **AFFIRMING CLIENT'S PERCEPTIONS**: The extent to which the coach affirms participants' self-awareness (e.g. thoughts, feelings, behaviors, and experiences).							
**1-LOW**	**2**	**3**	**4**	**5**	**6**	**7-HIGH**	**N/A**
☐	☐	☐	☐	☐	☐	☐	☐
6. **RETURNING THE FOCUS TO THE CLIENT**: The extent to which the coach encourages the client to return the focus to themselves and to possible solutions.							
**1-LOW**	**2**	**3**	**4**	**5**	**6**	**7-HIGH**	**N/A**
☐	☐	☐	☐	☐	☐	☐	☐
7. **AMPLIFYING SOLUTION TALK**: The extent to which the coach encourages the participant to discuss what aspects of his/her life they want to be different and the possibilities for making it happen.							
**1-LOW**	**2**	**3**	**4**	**5**	**6**	**7-HIGH**	**N/A**
☐	☐	☐	☐	☐	☐	☐	☐
