# Supplementary material for: Pediatric tele-coaching fidelity evaluation: Feasibility, perceived satisfaction and usefulness of a new measure
Source: Front Rehabil Sci. 2023 Feb 21;4:1057641. doi: 10.3389/fresc.2023.1057641 (PMC9989194; doi:10.3389/fresc.2023.1057641)
Supplement: Supplementary file 5 [file Image5.pdf]

Supplementary Material 5 CO-FIDEL Version 1.0 detailed description and rating procedure

Each section is scored separately, and the overall fidelity rating is determined as the average ± standard deviation (SD) of the 4 obtained scores

Section	*Method of scoring*	*Description*	*Equation*
**1) Overview: Coaching Delivery Content & Attitude**	10 parts, scored from 1 - "Low" to 7- "High".	Coach's ability to guide the participants through the content of each *BRIGHT Coaching* session, respecting the timeline and the related activities of each session, and the overall attitude of the coach during the session.	$\frac{\sum scores\ in\ each\ of\ the\ 10\ subsections}{70}\ x\ 100$
**2) Global Coach Ratings/Motivational Interviewing Skills Code (MISC)**	5 subsections, scored from 1 - "Low" to 7- "High".	Refers to motivational interviewing skills (e.g., acceptance, empathy, collaboration, evocation, and support of autonomy).	$\frac{\sum scores\ in\ each\ of\ the\ 5\ subsections}{35}\ x\ 100$
**3) Behavioral Counts/MISC**	Identifying the number of times coach uses consistent vs. inconsistent skills.	Refers to behavioural counts consistent with the motivational interviewing skill code (e.g., affirmation, reflection, reframing, advice with permission) vs. those inconsistent (e.g., confrontation, warning).	$\frac{\sum consistent\ \ MISC\ responses\ \ during\ session}{\sum consistent\ \ \&\ inconsistent\ \ MISC\ responses\ during\ session}\ x\ 100$
**4) Global Coach Ratings- Solution Focused Interview Skills**	7 subsections, scored from 1 - "Low" to 7- "High".	It pertains to the use of solution-focused interview skills (e.g., tolerating silences, complimenting, and amplifying solution talk).	$\frac{\sum scores\ in\ each\ of\ the\ 6\ of\ 7\ rated\ subsections}{42\ or\ 49}\ x\ 100$

Sections 2, 3, and 4 were designed to match the types of behaviors to be measured that are program-specific, essential, and behaviors that need to be avoided (Waltz, Addis, Koerner, & Jacobson, 1993). In addition, as recommended by previous research, we considered including frequency counts of particular behaviors (Waltz et al., 1993) (i.e., Section 3) and the use of a rating scale to better reflect rater's true evaluation (Preston & Colman, 2000) (i.e., Section 2)
