# Supplementary material for: Pediatric tele-coaching fidelity evaluation: Feasibility, perceived satisfaction and usefulness of a new measure
Source: Front Rehabil Sci. 2023 Feb 21;4:1057641. doi: 10.3389/fresc.2023.1057641 (PMC9989194; doi:10.3389/fresc.2023.1057641)
Supplement: Supplementary file 6 [file Image6.pdf]

Supplementary Material 6 CO-FIDEL use evaluation

	Not at all	Slightly	Moderately	Very much
**Overall, the extent to which are you satisfied with the CO-FIDEL's:**				
…content?	☐	☐	☐	☐
…format?	☐	☐	☐	☐
…administration frequency?	☐	☐	☐	☐
…understandability?	☐	☐	☐	☐
…comprehensiveness (complete/covers all important items)?	☐	☐	☐	☐
…relevance to your training needs as a coach?	☐	☐	☐	☐
…usefulness/helpfulness to you as a coach?	☐	☐	☐	☐
**Overall, the extent to which do you:**				
…feel assured/positive/motivated when reviewing your CO-FIDEL assessments?	☐	☐	☐	☐
…feel understood/supported by the lead coach when reviewing your CO-FIDEL assessment?	☐	☐	☐	☐
…feel that you benefit from the use of the CO-FIDEL?	☐	☐	☐	☐

Question 1: What do you most appreciate about the CO-FIDEL and why?

Question 2: What do you least appreciate about the CO-FIDEL and why?

Question 3: How do you think the CO-FIDEL influenced your coaching skills and the training process?

Question 4: What would you like to tell people involved in developing training protocols for coaches involved in programs such as BRIGHT Coaching?

Question 5: What else would you like us to know about your experience with CO-FIDEL and/or your training?
